# Philosophical Approach to Neural Autoantibodies in Psychiatric Disease—Multi-Systemic Dynamic Continuum from Protective to Harmful Autoimmunity in Neuronal Systems

**DOI:** 10.3390/antib12010001

**Published:** 2022-12-23

**Authors:** Niels Hansen

**Affiliations:** 1 Department of Psychiatry and Psychotherapy, University Medical Center Göttingen, 37075 Göttingen, Germany; niels.hansen@med.uni-goettingen.de; 2 Translational Psychoneuroscience, University Medical Center Göttingen, 37075 Göttingen, Germany

**Keywords:** immunopsychiatry, autoimmunity, autoantibodies, philosophy, biology

## Abstract

(1) Background: philosophical views are important to enable a general and multi-systemic view of the potential understanding of autoimmunity in psychiatric disease that is not solely reflected by an immunological viewpoint. (2) Methods: we reviewed current theories of autoimmunity. (3) Results: we propose a novel area view integrating the “self/non-self” and “continuity” model into the expression of varied forms of autoimmunity in psychiatric disease, ranging from protective to harmful autoimmunity consequences framed into micro-systems (nerve cells) and macro-systems (neuronal networks), termed the “multi-systemic dynamic continuum model”. (4) Conclusions: autoimmunity’s dynamic spectrum is delineated here as something that probably functions as a whole entity to maintain, first of all, human homeostasis in behavior affecting cells or neuronal networks differently, and secondly to prevent psychiatric disease.

## 1. Introduction—Immunological Conception of Central Nervous System Autoimmunity

The term central nervous system (CNS) autoimmunity refers to an immune reaction directed against the body´s own antigens within CNS structures from an immunological point of view. The immune reaction is so diverse and complex that it cannot be simplified as two main mechanisms. Autoantibody-associated psychiatric disease can, however, be categorized as two main immune mechanisms, although other important immune mechanisms also exist. One potential immune mechanism in autoimmune-mediated secondary psychiatric disorders that are associated with autoantibodies to intracellular antigens, is likely to be characterized predominantly by a T cell-dependent mechanism. This suggestion is based on the observation that in encephalitides associated with antibodies and intracellular antigens, CD8+ T cells were frequently found in encephalitic brain tissue [1]. The other major mechanism is characterized by the action of neural autoantibodies, such as antibodies against the N-methyl-D-aspartate receptor [2] detected in an autoimmune encephalitis of psychiatric phenotype [3]. Neural autoantibodies are part of the immune defense, which can play a protective role in destroying cancer (as paraneoplastic antibodies on the one hand), but they can also be destructive when accompanying CNS inflammation and neuronal nerve cell damage [4]. Neuronal autoantibodies may be associated with psychiatric disorders, although no evidence of brain inflammation or brain damage has been found. In such conditions, the significance of elevated levels of neural autoantibodies remains unclear according to what we know now. Specific biomarkers and clinical conditions with specific criteria, as formulated for autoimmune encephalitis [5], autoimmune psychosis [6], or autoimmune-based psychiatric syndrome [7], indicate the possible, or even probable, autoimmune origin of psychiatric symptoms in association with the detection of neural autoantibodies.

This review addresses, on the one hand, whether autoimmunity can be reduced to the distinction between an attack on the self as an autoimmune response and an attack on the non-self as an action that is not part of an autoimmune response. On the other hand, it may also be that autoimmunity, in particular, which has been found in psychiatric disorders probably mediated by autoantibodies, is more complex than the theory of the distinction between self and non-self. We favor the latter, and will make some arguments in our review. In our view, in addition to autoimmunity in psychiatric disorders (which may be evidenced by autoantibodies on the one hand, but may also be due to T cell-driven immune responses on the other), many other factors that may contribute to autoimmunity are important, such as T cells, regulatory T cells (Tregs), and B cells.

## 2. Methods

We searched PubMed for articles on theories of autoimmunity and autoantibodies related to psychiatric disorders. Our narrative review is based on the selection of these articles.

## 3. Results

We first introduce relevant theories of autoimmunity and then describe autoantibodies in psychiatric disorders as a possible manifestation of autoimmunity. Later, three different types of autoimmunity are presented, representing possible autoimmunity in autoantibody-associated psychiatric disorders. 

### 3.1. Philosophical Conception of CNS Autoimmunity

A philosophical consideration of CNS autoimmunity comprises two main models entailing explanations, namely the “self-model or non-self-model” as suggested by Burnet [8], and the “continuity hypothesis”, proposed by Pradeu and Carosella [9]. The “self/non-self-model” is currently one of the most common concepts for explaining CNS autoimmunity. This model is based on the assumption that foreign (non-self) elements trigger an immune reaction, whereas the self does not cause an immune reaction [10]. However, there are serious limitations associated with this model, as the immune system needs to include reactions against the self, i.e., if cancer cells develop. Furthermore, other immune cells such as (the much later discovered) regulatory T cells controlling other lymphocyte populations are not considered in this model [9]. Regulatory T cells as “self-cells” can be regarded as cells that respond to other self-cells, such as T-lymphocytes. Thus, immune cells can also be directed against “self” structures, resulting in an autoimmune regulation of immune cell populations. A model differing from the “self/non-self model”, has been developed, namely the “continuity model” [9]. The “continuity model” [9] states that the immune system serves to continuously distinguish antigenic epitopes present in the organism independently of their “self/non-self” nature. The continuity of immune interactions can be interrupted by novel epitopes. The “continuity” hypothesis represents a philosophical shift from a substance-related biological identity to a biological identity based on continuity [9]. The continuity model is suitable for addressing the biological identity of potential autoimmunity, but has limitations concerning the expression forms of CNS autoimmunity in psychiatric disease, addressed by this review below. Another development is the “discontinuity theory,” which states that immune responses depend on the discontinuity of the antigen [11,12]. This theory establishes general principles that are essential to creating a unified immune concept. Selected current concepts of CNS autoimmunity are delineated in Table 1.

### 3.2. Autoantibody-Mediated Psychiatric Disease

To understand the role of autoimmunity in psychiatric disease, we will elucidate the role neural autoantibodies play in psychiatric disease. Neural autoantibodies are increasingly detected in association with different psychiatric disorders, ranging from dementia to psychotic disorders [6,19,20,21,22]. The meaning of these neural autoantibodies is still enigmatic, although several models have postulated functions in aberrant synaptic transmission [23] and networking [24], as well as inducing specific psychiatric symptoms such as psychosis [25]. An autoimmune basis of psychiatric syndromes is fulfilled if specific criteria are present, such as (1) CNS inflammation measured by CSF pleocytosis, intrathecal IgG synthesis or other signs of brain inflammation in neuroimaging, in conjunction with (2) detecting autoantibodies in the serum and/or blood, (3) specific neuropsychiatric constellations termed as “autoimmune indicators”, such as seizures, motor abnormalities such as catatonia, fever, altered consciousness, severe cognitive dysfunction, or an adverse response to psychopharmacologic drugs [7]. By applying these criteria, clinicians can diagnose an autoimmune origin as being possible or definitive [7]. Autoantibody-mediated psychiatric disease can, on the other hand, originate from an organic autoimmune encephalitis when mainly relying on the Graus criteria [5]. Their criteria were slightly modified in a recent case series in which patients presenting primarily psychiatric symptoms were classified as psychiatric autoimmune encephalitis patients [21]. What is common to both classifications is the required occurrence of neural autoantibodies [5,7]. However, in the Hansen et al. [7] classification, the formulated criteria require signs of brain damage or inflammation via specific additional diagnostics to diagnose an autoimmune-originating psychiatric syndrome. In contrast, the presence in a recent case series of well-characterized autoantibodies such as LGI1, NMDAR or MOG antibodies [21] was regarded as a sufficient criterion to assume a probable psychiatric autoimmune encephalitis. Thus, there is substantial controversy at the moment about which diagnostic evidence must be present to classify a psychiatric disease as being autoimmune-based. We favor a more conservative approach to classifying a psychiatric syndrome as autoimmune-mediated. Several autoantibodies, such as those that are paraneoplastic, are not believed to contribute to the pathogenesis of the disease on their own, but rather in conjunction with T cells. The concept of autoimmunity in psychiatric disease probably encompasses two different mechanisms: (1) an autoantibody-mediated autoimmunity mechanism, mainly detected in patients with antibodies against membrane-surface autoantibodies, and (2), a T cell-mediated mechanism of autoimmunity detected in patients with antibodies against intracellular target antigens. Both mechanisms share an immunity process requiring the ability to have previously made a distinction between the group property “self” and its counterpart “non-self” or “foreign”. Disease states such as autoantibody-associated psychiatric disease incorporate the recognition of the “foreign” either as one’s own tissue or as microorganisms. This process is active and dynamic, so that the borders between the self and of identifying the “foreign” are not constant. The immune system is activated when “foreign” elements are recognized within the body. This recognition of the “foreign” involves a prior process of self-tolerance. The breakdown of self-tolerance induces an immune-system activation, enabling the self and non-self dichotomy, but it also leads to the continuous recognition of antigen epitopes. Such an immune-system activation can be “protective” or “aggressive”. The term protective autoimmunity refers to the autoantibodies and B cells responsible for autoantibody production in psychiatric autoimmune encephalitis or autoimmune-mediated psychiatric syndromes, but also to T cell pathology, as seen in patients with probable autoimmune-mediated psychiatric syndromes associated with intracellular autoantibodies, as described below. In our model presented in Section 3.3, Section 3.4 and Section 3.5, we establish a biological basis for psychiatric disorders that links neuronal systems to autoimmune processes. Autoinflammation in autoimmune states in organic psychiatric disorders implies minimal changes, such as mild encephalitis, and in brain tissue changes such as inflammation or brain injury.

### 3.3. Protective Autoimmunity

We found a protective autoimmunity with autoantibodies if the immune reaction takes place and eliminates “foreign” or “continuous” antigen epitope elements, such as a paraneoplastic action that causes the elimination of a tumor. Note that neural autoantibodies can be paraneoplastic in nature, such as intracellular antibodies, which means that neural autoantibodies can be the precursor or even consequence of cancer. Neural autoantibodies are often indicative of an unspecified immune response against tumor antigens sharing features with the targeted neural antigens. Thus, neural autoantibodies may be protective, as they are indications of a tumor immunity process taking place. Moreover, these autoantibodies may indicate a potentially harmful future, in that the appearance of these autoantibodies could act as the body’s alarm system before a potential cancer develops, and can thus be interpreted as protective.

**Figure 1:**
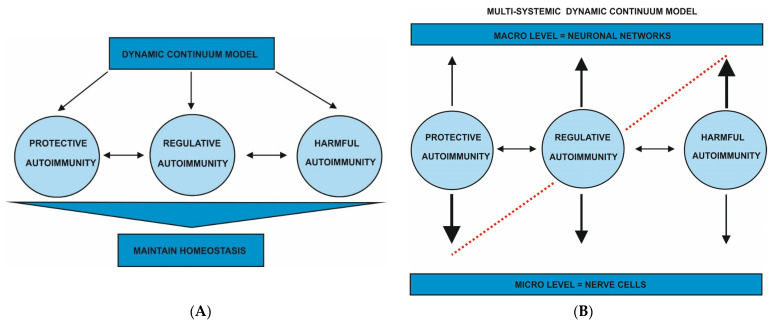
(**A**) Dynamic continuum model of autoimmunity. The dynamic continuum model of autoimmunity is depicted in Figure 1, and ranges from protective and regulatory to harmful forms of autoimmunity, which all serve to maintain brain homeostasis and thus mental health. (**B**) Dynamic continuum model of autoimmunity categorized into neuronal systems. The neuronal systems that are affected by the postulated three types of autoimmunity are the nerve cell on a micro level and neuronal networks on a macro level. Each type of autoimmunity differently affects these neuronal systems, so that a shift more affecting a micro level in protective autoimmunity can have an effect on the macro level with more consequences of harmful autoimmunity for neuronal networks can be observed (multi-systemic dynamic continuum model). The dashed line represents potential imbalance if one form of autoimmunity increases. For example, when harmful autoimmunity increases, protective autoimmunity decreases. When regulatory autoimmunity predominates, harmful and protective autoimmunity are equal. The increase in one form of autoimmunity is additionally indicated by the arrow’s size: a larger arrow indicates this form of autoimmunity’s predominance.

No symptoms are felt from the patient and the action leads to optimization of survival. The immune reactions are believed to be part of an active process that is regarded as a complex “normal” physiology [26]. It is known that natural autoantibodies exist that have protective roles and exist in normal [27] and also in disease conditions [28]. Protective autoimmunity is present to recognize epitopes that suggest potential damage for the body. Such a “healthy” autoimmunity protects the body from damage by cancer (Figure 1A). Protective autoimmunity is mainly based on micro-immune reactions on the cell level on the one hand, while on the other hand, protective immune reactions against antigenic structures in the nervous system (whose function is protective) may also affect neuronal networks on the macro level (Figure 1B). Thus, protective autoimmunity could help neuronal systems and neuronal tissue repair and restore themselves to maintain their previous physiological functions.

### 3.4. Regulatory Autoimmunity

Psychiatric symptoms coinciding with autoimmune-based psychiatric disease are potential correlates of CNS autoinflammation. Although such patients suffer from transient and reversible psychiatric symptoms, no relevant brain damage or brain inflammation is detected when conducting diagnostics such as EEG, CSF analysis, and MRI. The immunity process serves to regulate immunity against the self along with ongoing negotiation of the interaction between the self and “non-self”, as described by Tauber [29], or via continuous recognition of endogenous or exogenous epitopes, as proposed by Pradeu and Carosella [9]. Autoimmunity is part of a balanced physiologic process. It seems to be an action that regulates dynamic self-homeostasis. The regulatory T cells (Tregs) are a paradigmatic example of a regulatory autoimmune reaction to drive or limit cellular immune responses (Figure 1A). Tregs, such as Foxp3-expressing CD4+ Treg cells, are central to preventing the loss of self-tolerance so that autoimmunity states can be induced. In addition, these cells contribute to maintaining immune homeostasis [30]. Treg cells are not directly associated with autoantibodies. Treg cell functions that are not dependent on autoantibodies include the suppression of other T cells that dampen the immune or autoimmune response. Tregs may also limit CNS inflammation by secreting interleukin 10. Treg cells may have neuroprotective properties by placing microglia in a neuroprotective state [31]. In addition, there is a specific subset of Treg cells characterized by CD69+ that is increased in mice and humans and thought to control neuroinflammation [31]. When brain-resistant Treg cells are lost, neuroinflammatory states are enhanced. These specific Tregs are thus important in regulating autoimmunity in the CNS. Another function potentially relevant to Treg cells’ regulatory capability is their ability to downregulate calcium influx, a function impaired in patients with autoimmune CNS diseases [32]. On the other hand, Treg cells may also promote autoantibody production by producing neuritin via follicular Treg cells, which could thereby inhibit autoantibody production by B cells (B cell-driven autoimmunity) [33]. Thus, B cells producing autoantibodies are also an important aspect of regulatory autoimmunity. Moreover, regulatory autoimmunity plays a role in the clearance of cancer cells by cytotoxic CD8+ T cells without the aid of neural autoantibodies. The central role of cytotoxic CD8+ T cells in cancer cell clearance was recently confirmed in an animal study [34]. Taken together, regulatory autoimmunity could also help to reduce brain damage or inflammation in brain tissues already affected by autoimmunity processes.

### 3.5. Harmful Autoimmunity

Autoimmune processes in the CNS can cause severe damage and neuronal loss. Persistent psychiatric symptoms associated with neural autoantibodies might be a severe and harmful consequence of CNS autoimmunity (Figure 1A). Harmful autoimmunity should also be considered from a psychoneuroimmunological perspective. Anomalies in one cognitive system can affect other cognitive systems. Autoantibodies can enter the brain and induce a severe inflammation that can also trigger transient or persistent focal brain damage, such as hippocampal damage in LGI1 autoantibody-associated disease [4] or unspecific axonal brain damage indicated by elevated neurofilament light chains in the cerebrospinal fluid in LGI1 and NMDAR encephalitis [35]. Harmful autoimmunity, compared to regulatory or protective autoimmunity, is more relevant for diagnostics, as in conditions of harmful autoimmunity brain damage or brain inflammation can be found. The harmful type of autoimmunity ultimately affects both neural networks and nerve cells in a similar fashion and can be regarded as a shift from micro- to macro-neuronal systems (Figure 1B). Harmful autoimmunity thus has the most extensive consequences for neuronal systems compared with protective and regulatory autoimmunity. Harmful autoimmunity can result from both B or T cell-mediated immunopathology and B cell-directed autoantibodies, such as in membrane-surface autoantibody-based psychiatric autoimmune encephalitis. Thus, damaged autoimmunity means injury to the brain, and can also lead to severe inflammatory conditions therein and in associated neuronal systems.

## 4. Discussion

Our opinion article shows that autoimmunity based on autoantibodies may play different roles, ranging from physiologic homeostasis to regulatory processes in brain functioning and mental health, as well as building antibody-antigen complexes that might precede severe inflammation and brain damage. These varied expressions of autoimmunity serve to maintain brain homeostasis and are thus part of the “multi-systemic dynamic continuum model” (Figure 1B) in multiple neuronal systems ranging from the nerve cell to neuronal networks. According to this model, autoimmunity mainly evolves to promote brain homeostasis in a dynamic fashion to ensure the brain’s survival and mental health. Keeping these reflections in mind, it is obvious that harmful autoimmunity is also dedicated to maintaining brain function via different neuronal systems. Psychiatric autoimmune disease might therefore serve to help the individual re-adjust, as a manifestation of set-point modulation. Autoimmunity should be put in the context of evolutionary, neurocomputational, and cultural aspects to further investigate its effects in humans as protective or harmful. We believe that taking a philosophical approach helps us elucidate still-unanswered questions about the roles autoantibodies play in psychiatry. In a recent large-scale study [36], 49 different autoantibodies were sought in psychiatric disorders in over 7000 patients. In this study, autoantibodies were detected in 346 of 2043 patients with schizophrenia spectrum disorders and in 62 of 267 patients with affective disorders (suggesting a 17–23% frequency of autoantibodies in affective and psychotic disorders). However, autoantibodies in healthy controls were also detected in 400 of 2748 participants, suggesting a frequency of 15%. This large-scale study shows that autoantibodies are more prevalent in psychiatric disorders than in healthy controls, but autoantibodies per se may not be disease-defining because autoantibodies are also observed in healthy controls. Therefore, it is necessary that signs of CNS inflammation diagnosed via neuroimaging, CSF analysis or functional EEG help us to assess the relevance of these autoantibodies by classifying patient syndromes together with diagnostic findings into disease entities such as psychiatric autoimmune encephalitis [5,7,21] or autoimmune psychosis [6]. In another analysis by Daguano Gastaldi [36], immune checkpoint genotypes were identified as a determining factor for brain autoimmunity and the likelihood of disease development. Other factors may also play a role in determining whether autoantibodies are a relevant phenomenon in the expression of autoimmunity, or whether autoantibodies are merely indicative of brain injury following an immune response in a large proportion of patients. The latter is supported by the finding that a neurotrauma may predispose to a higher seroprevalence of NMDAR antibodies [36]. However, the production and occurrence of humoral autoimmunity is multifactorial, and cannot be proven by a single human autoantibody such as the NMDA antibody. Although the main role of autoimmunity is homeostatic regulation, it can also elicit concomitant psychiatric symptoms, which could in turn lead to a worse outcome. The homeostatic nature of autoimmunity is reflected in part by the relapsing-remitting character of autoantibody-associated psychiatric disorders, or it can even alleviate psychiatric disorders, unlike with progressing neurodegenerative diseases.

## Figures and Tables

**Table 1:** Current concepts of CNS autoimmunity—their advantages and disadvantages.

Theory	Advantage	Disadvantage	Reference
Self/non-self model	Differentiation of antigens depending on their “self/non-self” target	Does not consider biological identification based on continuity Does not include immune responses against cancer cells Regulatory T cells are not considered	[8]
Continuity model	Focus on biological identity instead of substance-related biological identity.	Self/non-self-antigen target not considered exclusively	[9]
Unified theory of brain pathologies	Loss of immunity to specific self-antigens or their inadequacy is considered an important factor for autoimmunity Good model for the occurrence of neurodegenerative diseases or psychiatric diseases Consideration of the specificity of CNS immunity	No consideration of autoantibody-mediated CNS pathology in psychiatric disorders with various forms of autoimmunity	[13]
Unifying model for immune network for CNS repair functions	Role of protective immune network is comprehensive and well considered	Other forms of autoimmunity, such as those which are deleterious or regulatory only, are disregarded	[14]
Inherent self-destructiveness	Both psychoanalytical and biological viewpoints are formulated in one concise concept Biological view of “self-reactivity” implies inherently self-destructive capacity Balance between constructive and pathological self-destruction A kind of “protective” autoimmunity is formulated	The death drive is too strong when assessed from a psychoanalytic viewpoint—too one-sided	[15]
Inside-out concept of autoimmunity	Autoimmunity is viewed as a physiological response to excessive antigen production in diseased tissue The response to antigens is predisposed	Genetic predisposition is only one factor—one-dimensional; multiple factors for predisposition are neglected Theory relies on multiple sclerosis data Model does not accommodate psychiatric disorders with autoantibodies (where tissue disease is much less prevalent)	[16]
Gateway theory	Link between neuronal and immune responses in the CNS concerning CNS autoimmunity Autoreactive T cells are considered Preventive form of autoimmunity considered	Limited to regional neuronal stimulators on blood vessels	[17]
Force of cognitive evolution	Beneficial factors of T cells on cognitive functions are considered Specific genes such as Foxp genes for Treg cells are considered Linkage between neuronal and T cells Balance control of proinflammatory autoreactive T cells is considered	The focus ignores autoantibody-mediated autoimmunity in psychiatric disease that is not primarily based on T cells	[18]
Multi-systemic dynamic continuum model	Considers biological identity instead of compound-related biological identity Considers different forms of autoimmunity, from protective to deleterious forms Considers both T and B cell-based pathology, Tregs, and autoantibodies in their diverse disease mechanisms Considers neuronal systems with specific forms of CNS autoimmunity Can be applied to autoantibody-mediated psychiatric disorders	Is not strictly limited to “self/non-self” determination of target antigens	

## Data Availability

No new data were created or analyzed in this study. Data sharing is not applicable to this article.
